# Complete mitochondrial genome of the endangered freshwater fish *Microphysogobio rapidus* (Cypriniformes, Cyprinidae) from Korea

**DOI:** 10.1080/23802359.2019.1704642

**Published:** 2020-01-08

**Authors:** Kang-Rae Kim, In-Chul Bang

**Affiliations:** Department of Life Science and Biotechnology, Soonchunhyang University, Asan, Korea

**Keywords:** *Microphysogobio rapidus*, Mitochondrial genome, Cyprinidae

## Abstract

*Microphysogobio rapidus* is an endangered freshwater fish from Korea with a limited distribution in the Nakdong River. Here, we determined the mitochondrial genome of *M. rapidus*, which consisted of 16,603 bp with 13 protein-coding genes, 2 ribosomal RNAs, 22 transfer RNA genes, and a control region (D-loop). The overall base composition of the complete genome was 29.96% A, 26.06% T, 17.24% G, and 26.74% C, with high A + T content of 56.02%.

*Microphysogobio rapidus* is an endemic freshwater species in Korea with a limited distribution in the Nakdong River. Its population is decreasing rapidly due to habitat destruction by human activity (Hong et al. 2017). In 2012, it was designated as a Korean endangered wild species class I (Ministry of Environment (ME) 2012). The complete mitochondrial genome was determined, providing basic data for conserving endangered species. A sample was collected from the Nam River, a tributary of the Nakdong River, and prepared for sequencing on an Illumina MiSeq instrument using the TruSeq Nano DNA kit (Illumina, San Diego, CA, USA). Extracted *M. rapidus* DNA is preserved at Soonchunhyang University, Republic of Korea (voucher number of SUC-7583).

The complete mitochondrial genome of *M. rapidus* (GenBank accession number MH713708) consisted of 16,603 bp, with 13 protein-coding, 2 ribosomal RNA (rRNA), and 22 transfer RNA (tRNA) genes and a control region (D-Loop). Except for the CO1 gene, which had a GTG start codon, the other 12 protein-coding genes (PCGs) started with ATG. The PCG ND1 terminated with TAG, a typical stop codon. Five PCGs (CO1, ATPase 8, ND4L, ND5, and ND6) were stopped with a complete ‘TAA,’ four PCGs (ND2, ATPase 6, ND3, and ND4) were stopped with an incomplete ‘TA-.’ Three PCGs (CO2, CO3, and Cytb) have an incomplete ‘T-,’ a common feature indicating the termination codon in *Microphysogobio* (Hwang et al. 2014; Park et al. 2016; Wang et al. 2016). The overall base composition of the *M. rapidus* genome was 29.96% A, 26.06% T, 17.24% G, and 26.74% C, with high A + T content of 56.02%. The ribosomal RNA (rRNA) consisted of 12S rRNA (959 bp) and 16S rRNA (1,690 bp). The control region (D-loop) was 926 bp in total length.

A molecular phylogenetic tree was constructed with the complete mitochondrial genomes of *Microphysogobio* species and the fish subfamily Gobioninae (Figure 1) using the maximum-likelihood method. In this study, *M. rapidus* was closely related to *M. yaluensis*.

**Figure 1.**
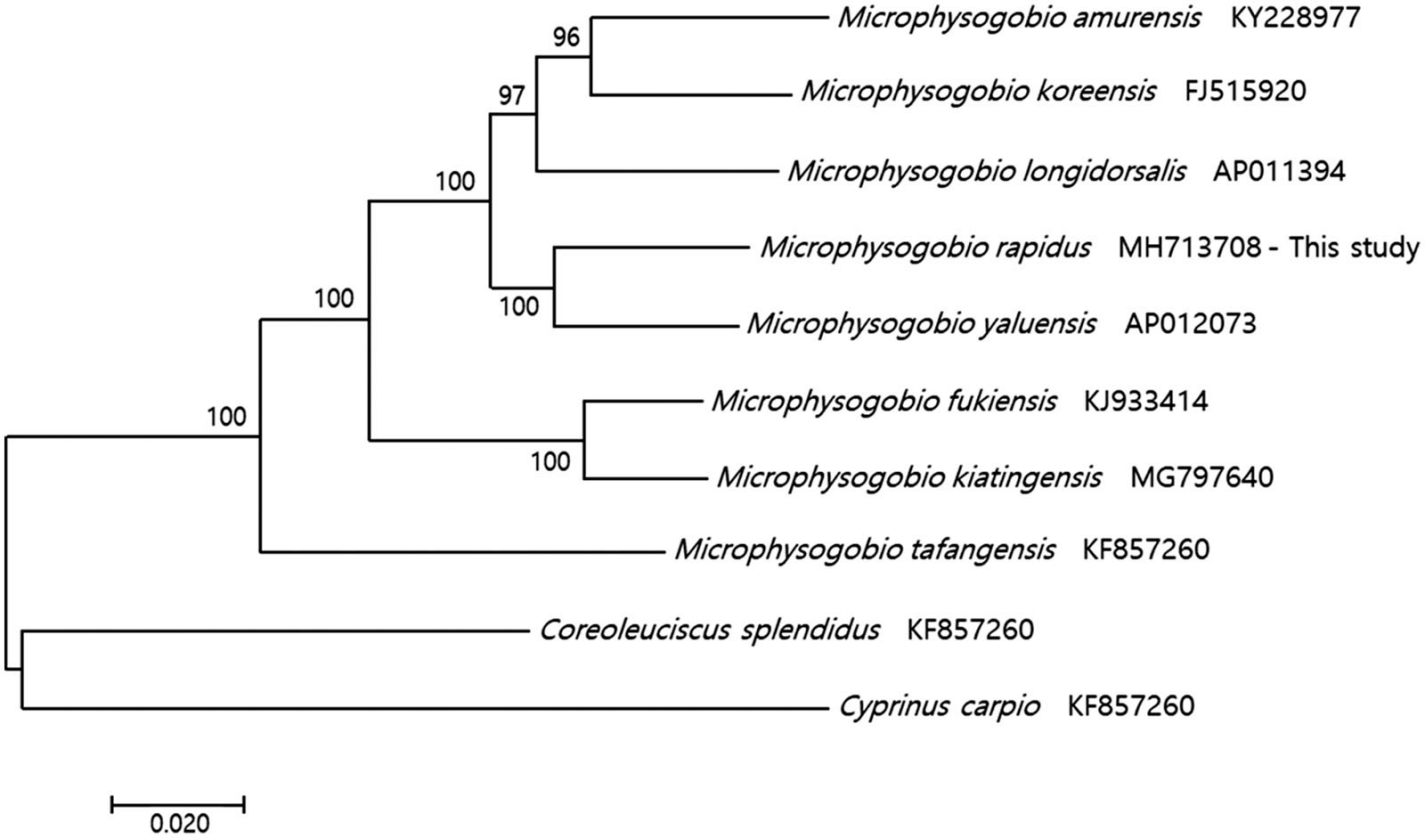
Molecular phylogenetic tree of the complete mitochondrial sequences of *Microphysogobio* species; the outgroup was the family Cyprinidae. The phylogenetic analysis used the maximum-likelihood method (1,000 bootstrap replicates), and the number at each node is the maximum-likelihood bootstrap proportion.
